# Supplementary material for: Diverse perceptual biases emerge from Hebbian plasticity in a recurrent neural network model
Source: Neuron. 2025 Nov 5;113(21):3673–3684.e6. doi: 10.1016/j.neuron.2025.09.037 (PMC12594053; doi:10.1016/j.neuron.2025.09.037)
Supplement: Document S1. Figures S1–S5 [file mmc1.pdf]

Neuron, Volume *113*

Supplemental information

Diverse perceptual biases emerge from Hebbian plasticity in a recurrent neural network model

Francesca Schönsberg, Davide Giana, Yukti Chopra, Mathew E. Diamond, and Sebastian Goldt

Supplemental Information

Diverse perceptual biases emerge
from Hebbian plasticity in a recurrent neural network model

Francesca Schönsberg, Davide Giana, Yukti Chopra, Mathew Diamond and Sebastian Goldt

Inventory of Supplemental Items

Supplementary Figure S1 (Related to STAR Methods section A.6) Supplementary Figure S1 represents the input pattern statistics and stimulation setup stochasticity.

Supplementary Figure S2 (Related to STAR Methods section B) Supplementary Figure S2 represents the excitatory/inhibitory switch in the effective connectivity.

Supplementary Figure S3 (Related to STAR Methods section D) Supplementary Figure S3 represents individual differences between random realisations of Experiment 2

Supplementary Figure S4 (Related to STAR Methods section E and F) Supplementary Figure S4 represents the effect of the variation of the model setup and parameters.

Supplementary Figure S5 (Related to STAR Methods section F.3) Supplementary Figure S5 represents the effective timescale of memory.

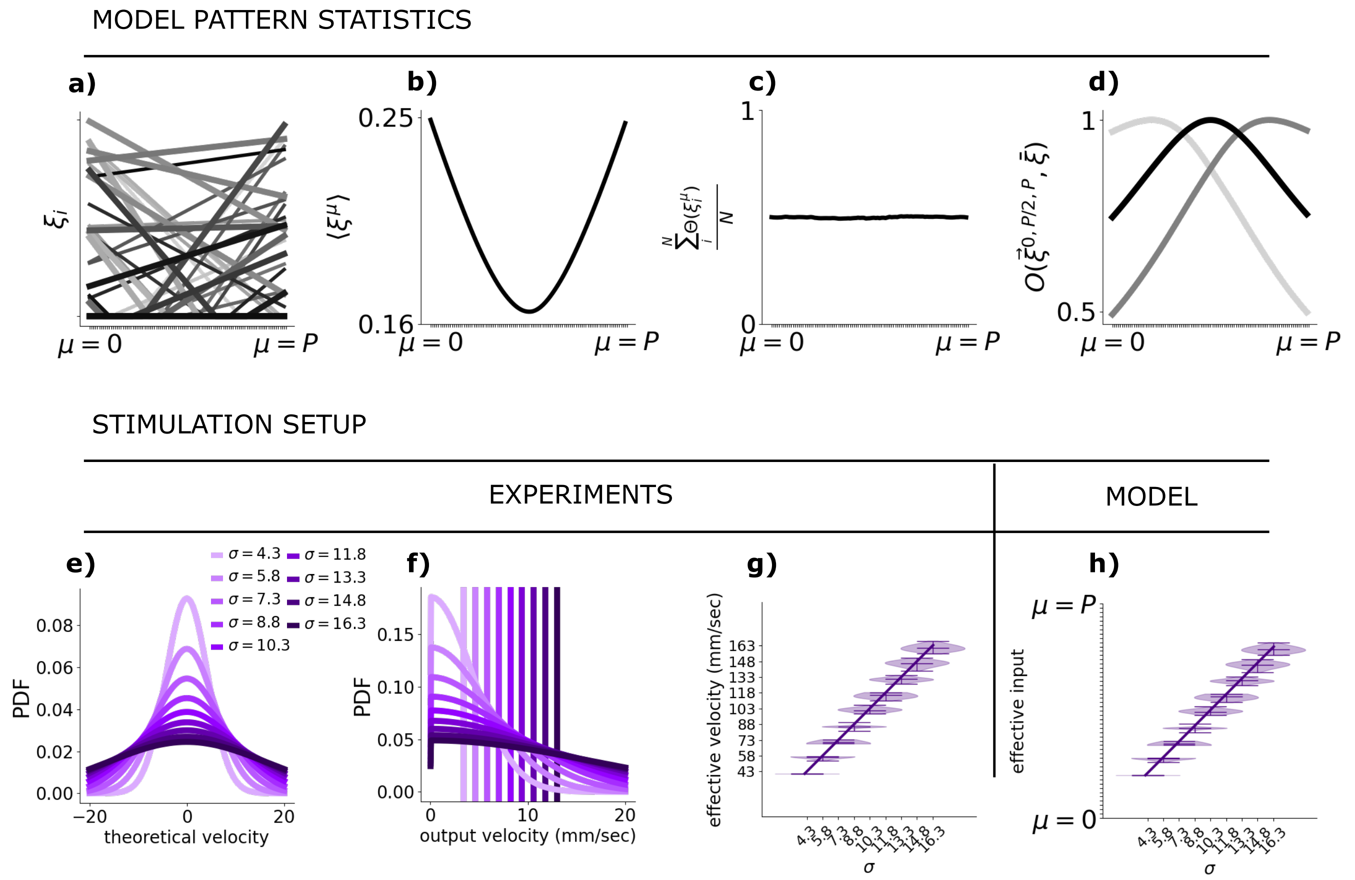

Supplementary Figure S1, related to STAR Methods section A.6

a) Fifty sample input profiles $\vec{\xi_i}$ (different colours) depicted for different intensities. **b)** Average activity across input profiles averaged over 100 realisations. **c)** Fraction of active units in the input profiles averaged over 100 realisations. **d)** Normalised overlap (cosine similarity) between $\vec{\xi}^{20}$ (light grey), $\vec{\xi}^{25}$ (black), $\vec{\xi}^{30}$ (dark grey), and all other patterns. **e)** Gaussian distribution with mean zero and standard deviation as labelled. **f)** Same as **e)**, but showing absolute values; the vertical line indicates the effective average activity. **g)** Violin plot representing the actual average obtained by sampling 5000 random numbers from the distributions in **f)**. **h)** Identical to **g)**, but with the input intensities discretised into P bins, illustrating the transition between experiments and model.

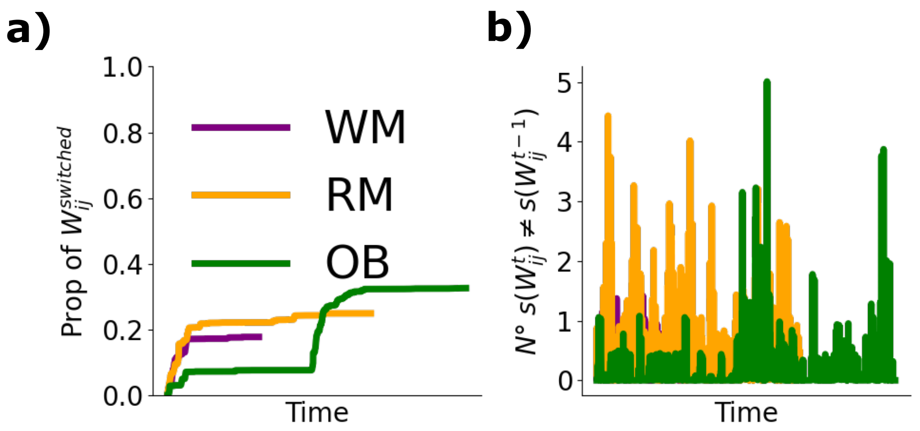

Supplementary Figure S2, related to STAR Methods section B

a) Proportion of weights that have made at least one switch at the different stages of the trial and **b)** Absolute number of weights which have changed sign from one step to the other for the three examples shown in Fig. 6 of the main text. Specifically one per paradigm, labelled with different colour. Purple: Working memory (WM); Orange: Reference memory (RM); Green: One-back (OB). Please note that the time is non matching due to the different length of the trials. In **b** one should consider the absolute number of changes in comparison with the total amount of weights which is N^2, with $N = 200$

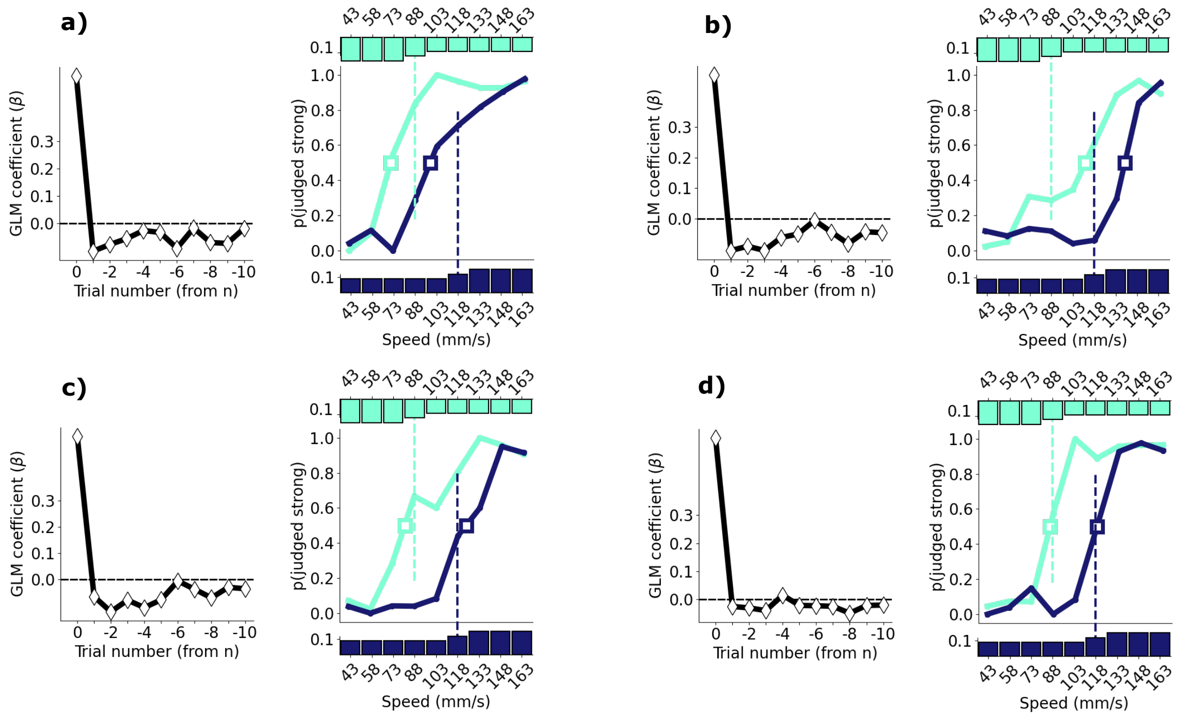

Supplementary Figure S3, related to STAR Methods section D

a)–d) Left, GLM as in fig. 4h. Right, psychometric curves as in fig. 4f (note that here the solid line is just joining the experimental points and is not a fit). Differently from fig. 4e–h we plot the results from 4 individual random realisations and not the average over 200 realisations.

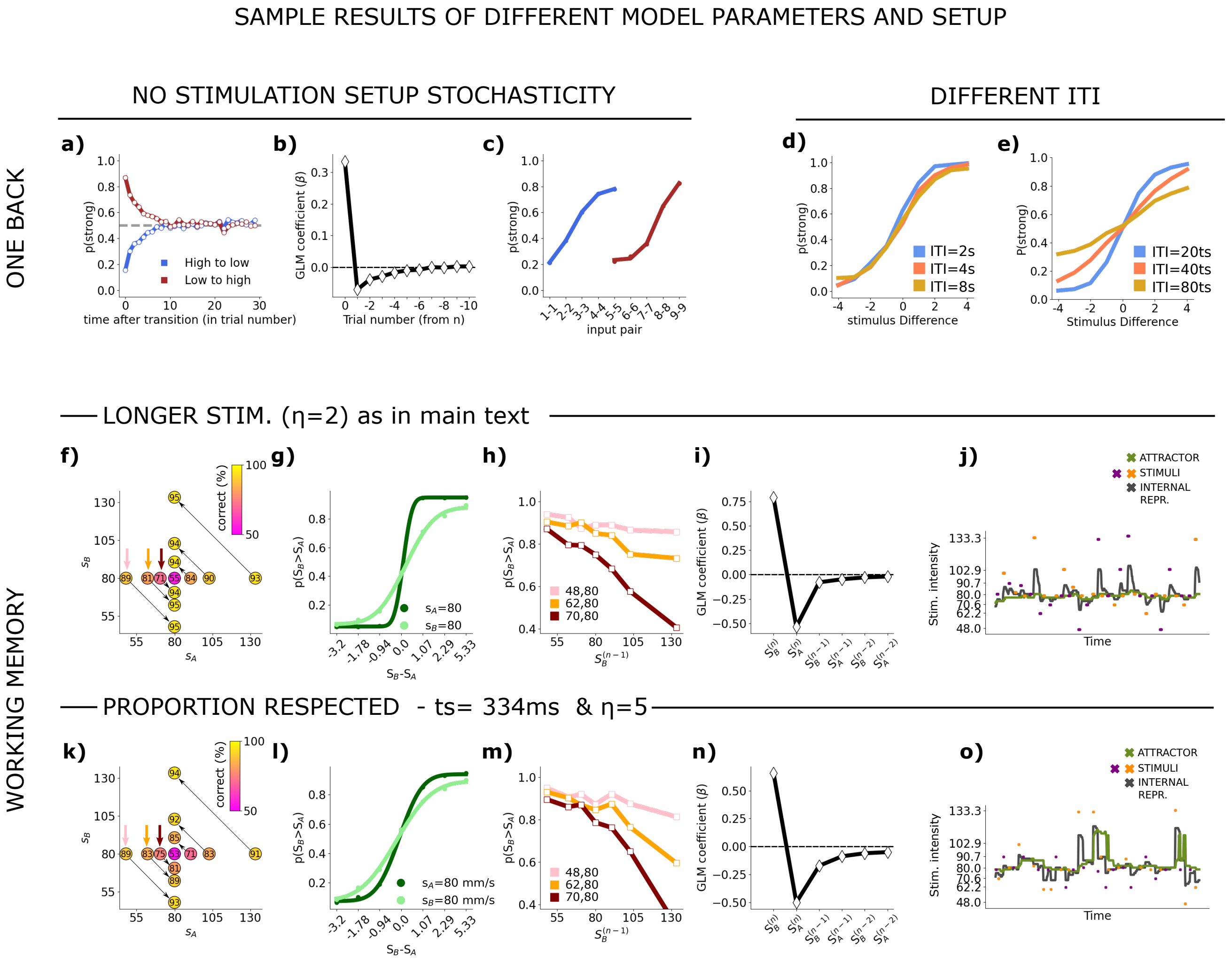

Supplementary Figure S4, related to STAR Methods section E and F Variation of model setup and parameters: **a–c** One-back Model without Input Stochasticity: The model and figures remain consistent with those reported in **??**e-g). The difference lies in the effective inputs, where the stochastic component of the stimulation setup is removed. **d–e** Time steps in our model: Example from the One Back paradigm. **d)** Conditioned psychometric curves based on the time elapsed between two stimuli (independent of the cloud). **e)** Same as **d)**, but for the model results. **f–q** Our model reproduces contraction bias and history-dependence of choices in the working memory experiment when the ratio between stimulus duration and ISI in simulations matches the ratio in the experiment. **f–o)** reproduce the panels of Figs. 2 e–h and Fig. 6 a) of our manuscript and are obtained from simulations. **f–j)** show the same plots obtained in a simulation where the ratio between inter-stimulus interval and stimulus duration matches the experiment. We also increased the stimulus intensity to $\eta = 5$. Averages are taken over 100 simulations, each consisting of 500 trials.

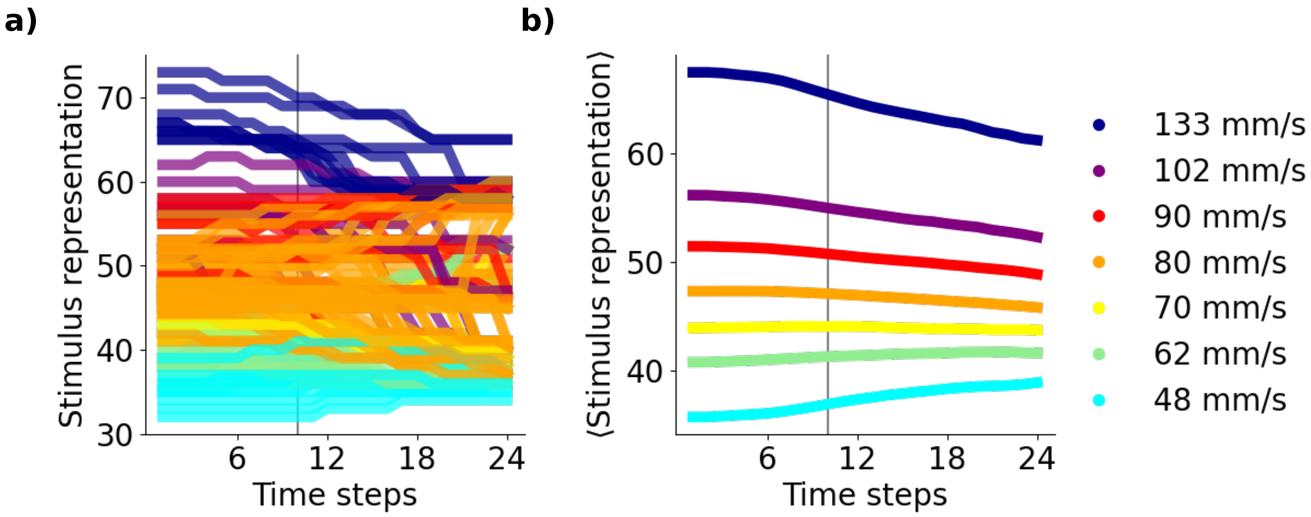

Supplementary Figure S5, related to STAR Methods section F.3

In a working memory paradigm with a model realization (using parameters from fig. 8k)–o), second row, where ISI and stimulus duration align with experiments), we analyse trials with a 24-time step ISI. a) Shows individual trials, where memory decay varies due to prior stimuli and the neural state before S_A. b) Displays the average across trials and realizations. The vertical line at 10 time steps indicates neuronal decay, yet memory persists beyond this point due to the attractor dynamics.
